# Experimental Investigation on Flexural Behavior of Desert Sand Concrete Beams Subjected to Freeze–Thaw Cycles

**DOI:** 10.3390/ma19122437

**Published:** 2026-06-07

**Authors:** Meng Wu, Zhiqiang Li, Yingsheng Dang, Feng Ji, Chao Huang, Jian Li

**Affiliations:** 1 College of Water Conservancy & Architectural Engineering, Shihezi University, Shihezi 832003, China; 17799937123@163.com (M.W.); lijian8.14@163.com (J.L.); 2 College of Mathematics and Physics, Xinjiang Institute of Engineering, Urumqi 830023, China; 3 College of Safety Science and Engineering, Xinjiang Institute of Engineering, Urumqi 830023, China

**Keywords:** desert sand concrete, freeze–thaw cycles, flexural performance, cracking moment, ultimate load-bearing capacity

## Abstract

To mitigate the shortage of natural river sand in northwest desert regions, utilize local desert sand resources, and address structural performance under harsh winter conditions, this study investigates the flexural behavior of freeze–thaw conditioned desert sand concrete beams (DSCBs) through rapid freeze–thaw and flexural testing. The investigated variables included desert sand replacement ratios (0%, 20%, 40%, 60%) and freeze–thaw cycles (0, 25, 50, 75). Failure modes, load–concrete strain curves, load–deflection relationships, and load–longitudinal reinforcement strain were analyzed. The results indicate that the crack development and failure modes of DSCBs are similar to those of normal concrete beams, and the plane-section assumption remains valid after freeze–thaw cycles. After 75 freeze–thaw cycles, specimens with the same replacement ratio exhibited the poorest mechanical properties—compared to unfrozen specimens, the ultimate capacity decreased by up to 17.5%, reinforcement strain increased by up to 31.9%, and failure deflection decreased by up to 62.0%. Under all freeze–thaw conditions, the 20% replacement ratio yielded the best performance, with ultimate capacity up to 5.3% higher, reinforcement strain up to 18.2% lower, and failure deflection up to 37.5% higher than those of ordinary concrete beams. Finally, correction factors for desert sand replacement ratio and freeze–thaw cycles were introduced to establish predictive equations for cracking moment and ultimate flexural capacity. The predictions are in good agreement with experimental results, providing a theoretical basis for engineering applications.

## 1. Introduction

With the ongoing progression of urbanization, the disequilibrium between the supply and demand of construction sand has grown increasingly acute [1]. Against this backdrop, the efficient and rational utilization of alternative resources has become a central research theme within the field. Northwestern China possesses abundant desert sand resources; however, desert sand differs significantly from natural river sand in terms of particle morphology, gradation characteristics, and surface properties. Compared with natural river sand, which is characterized by angular particles, reasonable gradation, and rough surfaces, desert sand particles generally exhibit higher roundness and sphericity, narrower particle size distribution, poorer gradation, and smoother surfaces. In addition, desert sand usually contains higher amounts of fine powder and soluble salts. These characteristics may weaken the bonding performance of the interfacial transition zone (ITZ) between aggregates and cement paste, reduce particle packing density, and increase internal porosity, thereby adversely affecting the freeze–thaw resistance of concrete. Previous studies have shown that the deterioration of concrete under freeze–thaw conditions is closely related to the initiation, propagation, and coalescence of internal microcracks. The interfacial transition zone is usually a critical region for preferential crack development and damage accumulation [2]. Therefore, for desert sand concrete, the influence of its unique particle characteristics on freeze–thaw damage evolution and structural mechanical behavior deserves further investigation. If used as a fine aggregate to replace river sand in concrete engineering [3,4], desert sand can not only help alleviate the shortage of natural river sand, but also promote the rational utilization of regional resources, which is of great significance for engineering applications and resource development [5,6].

However, due to the geographical distribution of deserts in China, concrete structures in desert regions are exposed to complex service environments, where durability issues caused by freeze–thaw cycles are particularly severe [7,8,9,10]. In recent years, extensive studies have been conducted both domestically and internationally on the mechanical behavior of desert sand concrete (DSC) exposed to freeze–thaw environments. Liu et al. [5] investigated the frost resistance of DSC and reported that freeze–thaw cycles lead to a degradation of its mechanical properties. Xi et al. [11] studied the degradation law of DSC mechanical properties under freeze–thaw cycles and established a corresponding damage evolution model. Li et al. [12] compared the durability of DSC and sandy concrete exposed to cyclic freeze–thaw actions and established a life prediction model based on Weibull distribution theory. Li et al. [13] prepared C30 concrete using desert sand mixed with tuff gravel as fine aggregate, established a prediction model based on the Weibull distribution through freeze–thaw performance tests. Bai et al. [14] systematically investigated the influence mechanism of different desert sand contents on frost resistance and established macro–micro relationships between compressive strength and porosity characteristics.

In addition, research on the structural behavior of DSC components has also achieved remarkable advances. Li et al. [15] investigated the flexural behavior of DSC beams (DSCBs), focusing on the effect of desert sand replacement ratio (0–80%) and derived a model for calculating the flexural capacity. Zhang et al. [16] studied the bending response of desert sand concrete-infilled steel tubular sections members and developed a refined numerical simulation framework for parametric assessment. A design formula for the flexural capacity of DS-CFST members was proposed, and the finite element results agreed well with experimental data. Pan et al. [17] investigated the flexural behavior of steel fiber-reinforced DSCBs and clarified the fracture mechanism through numerical analysis of strain patterns, crack propagation behaviors, and displacement contours. Li et al. [18] investigated the shear performance of DSCBs and constructed a computational model for the shear capacity of specimens. Li et al. [19] studied the axial compressive behavior of DSC short columns and established a strength calculation formula. Li et al. [20] conducted low-cycle loading tests on DSC columns and systematically studied the influences induced by shear span ratio, desert sand replacement ratio (up to 60%), axial compression ratio and stirrup ratio affecting seismic performance. A design model was established to predict the flexural and shear strength of DSC columns.

At present, most studies on the mechanical properties of DSC after freeze–thaw cycles remain at the material level, and even studies at the structural level are conducted under normal environmental conditions. However, studies on the flexural behavior of DSCBs after freeze–thaw cycles remain insufficient. In particular, limited attention has been paid to crack propagation behavior, the applicability of the plane section assumption, and the modification methods for the formulas specified in the current code [21]. This has restricted the promotion and application of DSC in cold-region engineering to some extent. Therefore, in this study, the desert sand replacement ratio and the number of freeze–thaw cycles were selected as variables to conduct flexural performance tests on DSCBs subjected to freeze–thaw cycles. The effects of these two factors on the deformation behavior and failure characteristics of DSCBs were analyzed, and the applicability of the plane section assumption was verified. Furthermore, based on the current Chinese Code for Design of Concrete Structures [21], calculation formulas for the cracking moment and ultimate flexural capacity of DSCBs under freeze–thaw conditions were established, providing a theoretical reference for the engineering application of DSC.

## 2. Materials and Methods

### 2.1. Material Properties and Mix Proportion Details

**Figure 1:**
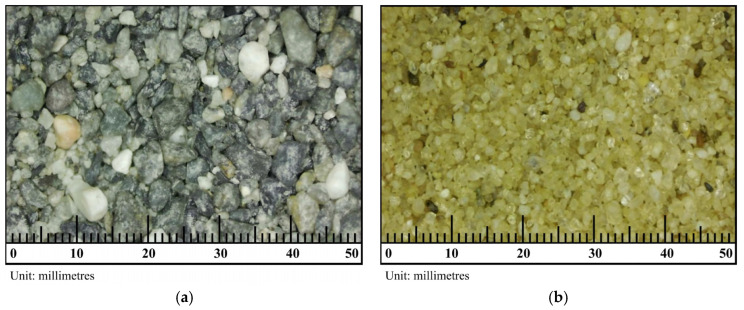
Apparent morphology of fine aggregate: (**a**) river sand; (**b**) desert sand.

**Figure 2:**
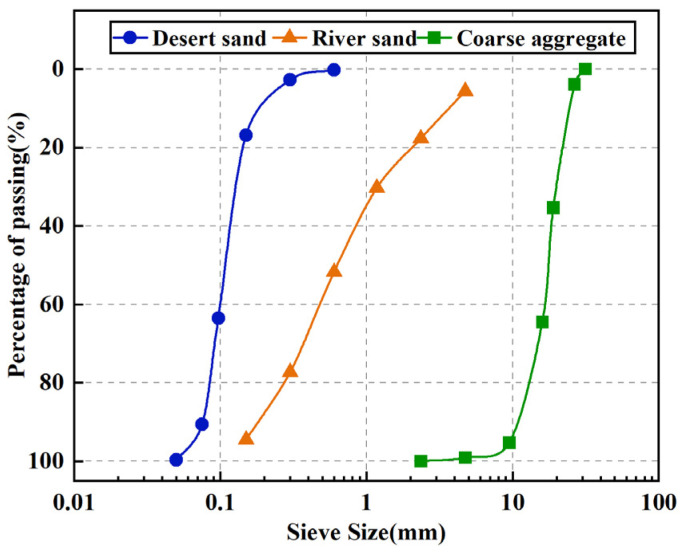
Aggregate grading curve.

The cement, desert sand, crushed stone, river sand, fly ash, water-reducing agent, water, and mix proportions used in this experiment were based on Ref. [19], and the detailed mix proportions are presented in Table 1. The main physical properties of the fine aggregate are listed in Table 2. The apparent morphology of the fine aggregate is shown in Figure 1. The particle size distribution grading curve of the aggregate used in the experiment is presented in Figure 2. During the casting of each specimen, three cube specimens with dimensions of 150 mm × 150 mm × 150 mm and three prism specimens with dimensions of 150 mm × 150 mm × 300 mm were reserved, which were used to determine the cubic compressive strength *f*_cu_ and axial compressive strength *f*_c_, and the cube and prism specimens were cured under the same conditions as the test specimens [21]. The measured mechanical properties of the concrete specimens are shown in Table 3. HRB400 grade steel bars (manufacturer: Xinjiang Bayi Iron & Steel Co., Ltd., Urumqi, China) were used, and their mechanical properties are presented in Table 4.

### 2.2. Specimen Design

**Figure 3:**
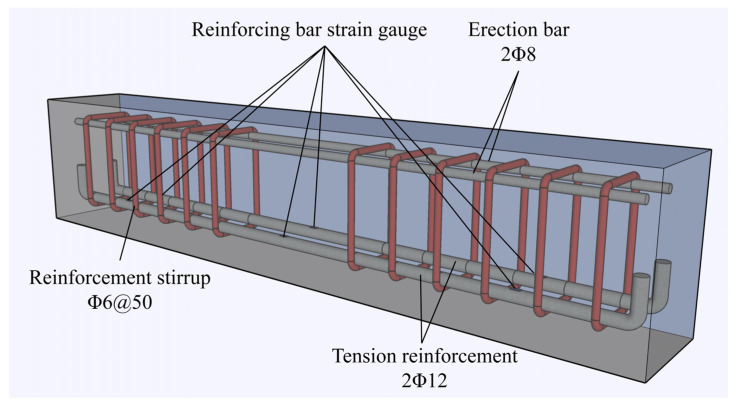
Schematic diagram of reinforcement and strain gage layout.

The experiment involved the preparation of 16 rectangular specimens with cross-sectional parameters of *b* × *h* = 100 mm × 150 mm. They were labeled as DSCB-M-R, where M = 1, 2, 3, 4 represents freeze–thaw cycles of 0, 25, 50, and 75, and R = 0, 20, 40, 60 indicates desert sand replacement rates of 0%, 20%, 40%, and 60%, respectively. The concrete design strength was set to C40, and all reinforcing bars in the specimens were HRB400-grade. The longitudinal reinforcement consisted of two Φ12 bars, the stirrups were Φ6 bars spaced at 50 mm, and the thickness of the protective cover was 20 mm. The specific parameters of the specimens are shown in Table 5 and Figure 3.

### 2.3. Experimental Loading and Testing Details

#### 2.3.1. Freeze–Thaw Cycle Testing

According to the rapid freezing method specified in the Standard for Test Methods of Long-term Performance and Durability of Ordinary Concrete [22], freeze–thaw cycle tests under water freezing and water thawing conditions were conducted using a TDRF-1 rapid freeze–thaw testing machine (Tianjin Huida Experimental Instrument Co., Ltd., Tianjin, China). During the freeze–thaw cycles, the liquid level in the specimen chamber was consistently maintained at 5 mm above the top surface of the specimens. Each freeze–thaw cycle lasted 2–4 h, with the thawing duration accounting for at least one quarter of the total cycle time. The test required that the minimum temperature at the center of the specimen be −18 °C ± 2 °C and the maximum temperature be 5 °C ± 2 °C. However, considering that the temperature-measuring specimen was a standard specimen, which is more sensitive to temperature variations and positioned near the side of the chamber, the maximum and minimum temperatures in this test were set to −20 °C ± 2 °C and 7 °C ± 2 °C, respectively. After 0, 25, 50, and 75 freeze–thaw cycles, the specimens were removed, cleaned of surface debris, and dried before performing surface evaluation.

#### 2.3.2. Bending Performance Test of Beams

**Figure 4:**
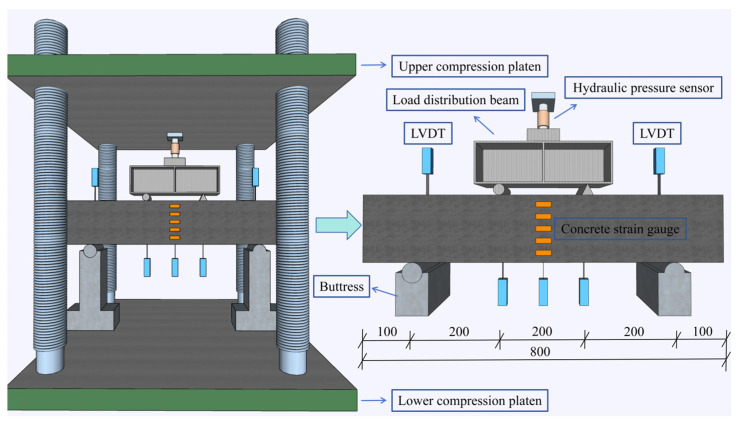
Test loading device and measuring point layout (Unit: mm).

The test was conducted using a 5000 kN long-column press (manufacturer: Changchun Testing Machine Co., Ltd., Changchun, China) to apply four-point bending loads, as shown in the loading configuration of Figure 4. To measure the strain in the longitudinal reinforcement, three sets of strain gauges were symmetrically placed between the mid-span, loading locations and bearing points, as exhibited in Figure 3. To detect concrete strain, five strain gauges were evenly arranged along the mid-span beam height. To measure deflection, three displacement meters were positioned at mid-span, while two others were located at the supporting points, as exhibited in Figure 4.

Loading on the specimens was conducted following the “Standard for Testing Methods of Concrete Structures” [23]. The pre-loading phase involved applying a 5 kN load in three stages. The formal loading phase began with a 10% increment of the ultimate load, switching to 5% increments once 90% of the ultimate load was reached. Crack patterns and widths were recorded during each loading stage until specimen failure. The experimental data were automatically collected using a TDS-530 data acquisition system. The primary data collected included concrete strain, longitudinal reinforcement strain, and beam deflection.

## 3. Experimental Outcomes and Analysis

### 3.1. Surface Condition of DSCB

**Figure 5:**
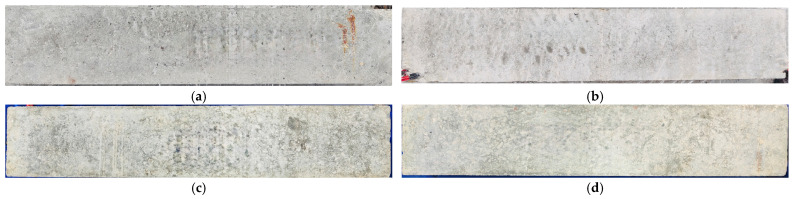
Apparent morphology of beams with different desert sand replacement ratios: (**a**) DSCB-2-0; (**b**) DSCB-2-20; (**c**) DSCB-2-40; (**d**) DSCB-2-60.

**Figure 6:**
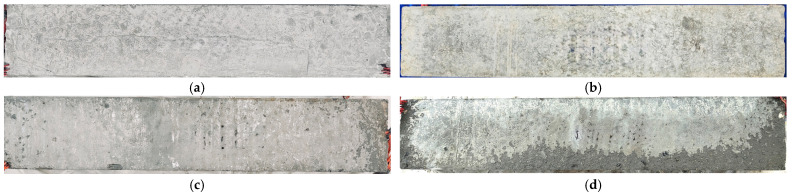
Apparent morphology of beams with varying freeze–thaw cycles: (**a**) DSCB-1-40; (**b**) DSCB-2-40; (**c**) DSCB-3-40; (**d**) DSCB-4-40.

Figure 5 and Figure 6 show the changes in the surface appearance of DSCBs after undergoing freeze–thaw cycles. As exhibited in Figure 5 (*n* = 25), no notable distinction can be found in the surface features of DSCBs with different *r* values under the same *n*.

As shown in Figure 6 (specimens with a desert sand replacement ratio of 40%), under the same replacement ratio *r*, the apparent morphology of DSCBs deteriorated significantly with the increase in the number of freeze–thaw cycles *n.* Surface pitting, mortar spalling, coarse aggregate exposure, and even aggregate detachment gradually appeared, while the damage at the corners and surrounding areas of the beams became more severe. The fundamental reason for this phenomenon is that, during freeze–thaw cycles, the water within the concrete pores repeatedly freezes and melts, generating considerable frost heaving pressure. When this pressure exceeds the tensile strength of concrete, a large number of internal microcracks are generated within the material [2,24]. As the number of freeze–thaw cycles increases, these microcracks continue to propagate and gradually coalesce, forming an internal damage network. This process leads to the deterioration of the interfacial transition zone (ITZ) and further induces macroscopic damage phenomena such as surface mortar spalling and aggregate exposure [2,24]. Specifically, when *n* = 0, the surface of the DSCBs remained smooth and intact. When *n* = 25, several slight mortar spalling areas could already be observed on the DSCB surface. When *n* = 50, mortar spalling became significantly more severe, and obvious pitting damage appeared in some areas, mainly concentrated near the beam ends and corners. When *n* = 75, large-area mortar spalling occurred on the DSCB surface, and coarse aggregates detached locally, forming obvious local spalling zones at the beam ends and lower regions of the specimens. From the perspective of material damage evolution, the slight spalling observed during the early freeze–thaw stage indicates that microcracks were still in the local initiation stage. As the number of freeze–thaw cycles increased, the occurrence of pitting and large-area spalling suggested that the microcracks had gradually evolved from local propagation into interconnected damage, significantly aggravating the deterioration of the internal material structure [2,25].

### 3.2. Cracking Distribution and Destruction Mode

**Figure 7:**
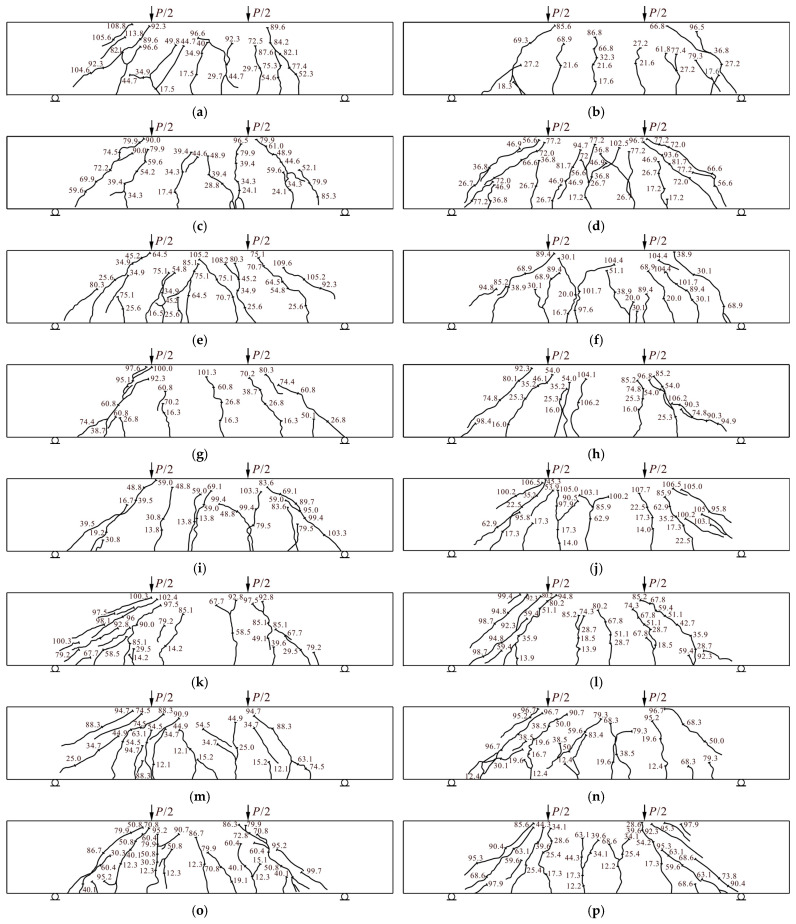
Crack development (Unit: kN): (**a**) DSCB-1-0; (**b**) DSCB-1-20; (**c**) DSCB-1-40; (**d**) DSCB-1-60; (**e**) DSCB-2-0; (**f**) DSCB-2-20; (**g**) DSCB-2-40; (**h**) DSCB-2-60; (**i**) DSCB-3-0; (**j**) DSCB-3-20; (**k**) DSCB-3-40; (**l**) DSCB-3-60; (**m**) DSCB-4-0; (**n**) DSCB-4-20; (**o**) DSCB-4-40; (**p**) DSCB-4-60.

Figure 7 illustrates the distribution of cracks and final failure patterns of the specimens. As shown in Figure 7, the crack propagation behavior and the failure characteristics of DSCBs resemble those of ordinary concrete beams. At the initial stage of loading, the specimens remain in the elastic state with no noticeable external changes. When the load reaches 11.3–14.9% of the ultimate load, vertical cracks first develop at the soffit of the pure bending zone, with lengths ranging from 28 to 68 mm and relatively small widths. When the load reaches 17.9–33.2% of the ultimate load, new cracks continuously form in the pure bending zone, with increasing width and height, while a few inclined cracks appear in the bending–shear zone and develop slowly. When the load reaches 34.4–65.5% of the ultimate load, inclined cracks develop rapidly and their widths increase significantly. When the load rises to 76.1–91.4% of its ultimate value, the development of cracks becomes relatively steady, while the widths continue to increase. The concrete in the compressive zone crushes at ultimate load, and the specimen subsequently fails.

It is noteworthy that the desert sand replacement ratio has a significant influence on crack patterns. Taking the group subjected to 75 freeze–thaw cycles as an example, the crack spacing of ordinary concrete beams is 65–85 mm, with a maximum crack width of 0.32 mm; whereas for the DSCBs with a 20% desert sand replacement ratio, the crack spacing decreases to 45–60 mm and the maximum crack width reduces to 0.21 mm, exhibiting a “multiple fine cracks” characteristic, indicating that an appropriate amount of desert sand can optimize crack distribution.

### 3.3. Plane Section Assumption

**Figure 8:**
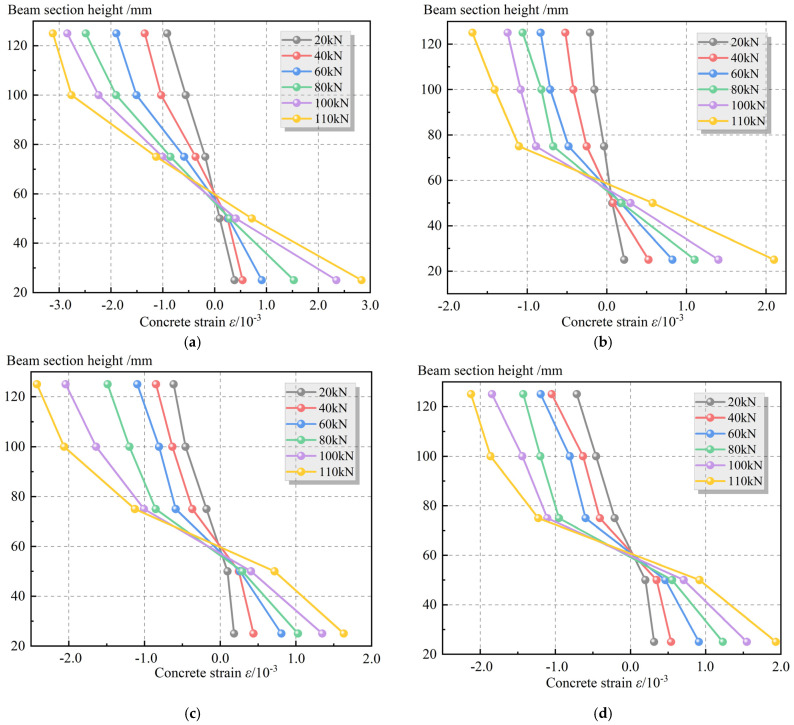
Concrete strain curves: (**a**) *r* = 0%; (**b**) *r* = 20%; (**c**) *r* = 40%; (**d**) *r* = 60%.

Figure 8 takes a group of specimens with *n* = 25 as an example to illustrate the variation in the distribution of concrete strain along the sectional height at the mid-span of DSCBs under various *r* values. As can be seen from the figure, under all loading levels, the strain distributions of concrete in the tension and compression zones of DSCBs are basically linearly distributed along the section height. That is, the strain values at measuring points located at different heights are approximately linearly related to their distances from the neutral axis. This indicates that the plane section hypothesis is still applicable to DSCBs after freeze–thaw cycles.

The plane section assumption is a fundamental premise for analyzing the flexural capacity of reinforced concrete beams, which establishes that the strain distribution along the section height is linear, thereby enabling the derivation of capacity calculation formulas based on geometric compatibility conditions, material constitutive relations, and internal force equilibrium. Verifying that the DSCB still satisfies the plane section assumption after freeze–thaw cycles is of significance in two aspects. First, it indicates that although freeze–thaw damage degrades the material properties of concrete, it does not alter the fundamental law of strain distribution across the section, and thus theoretical analysis can still be conducted based on this assumption. Second, it provides a theoretical basis for establishing the modified formulas for cracking moment and ultimate bearing capacity of DSCBs under freeze–thaw conditions in Chapter 3—only when the plane section assumption holds can the geometric relationships in the current Chinese Code for Design of Concrete Structures [21] be applied to derive modification coefficients for freeze–thaw damaged members through strain compatibility conditions.

### 3.4. Analysis of Load–Midspan Deflection Curves

#### 3.4.1. Effect of Desert Sand Replacement Ratio

**Figure 9:**
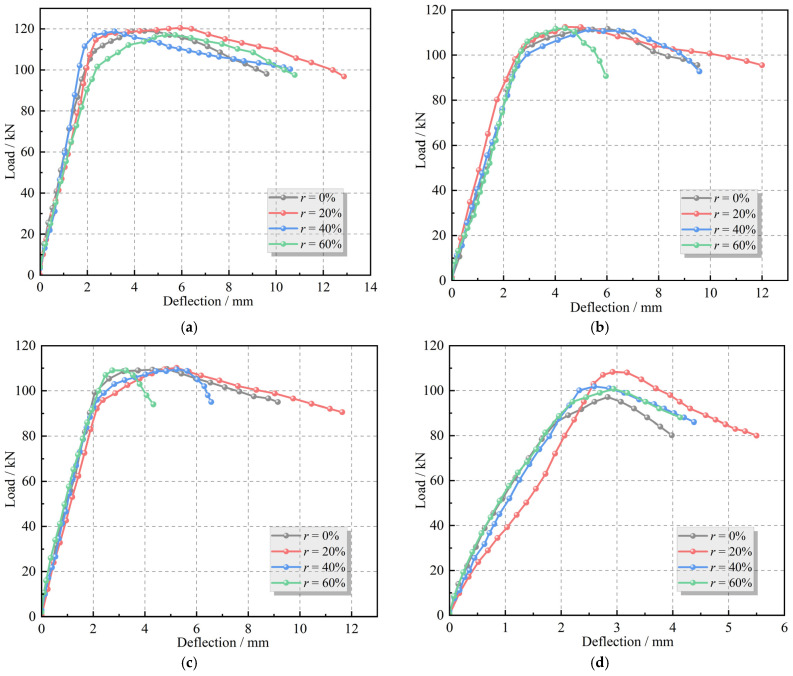
Load–deflection curves with varying desert sand replacement ratios: (**a**) *n* = 0 cycles; (**b**) *n* = 25 cycles; (**c**) *n* = 50 cycles; (**d**) *n* = 75 cycles.

Figure 9 presents the load–deflection curves of DSCBs with different desert sand replacement ratios. In general, at the initial loading stage, the concrete remains uncracked, and the specimens are in the elastic stage, with the curve showing an approximately linear relationship. After reaching the cracking load, cracks initiate at the bottom of the beam and propagate upward, leading to a reduction in beam stiffness and a decrease in the slope of the curve. After the reinforcement yields, the specimen enters the plastic stage, the slope of the curve further decreases, and the deflection increases more rapidly. At the ultimate load, the concrete in the compression zone is gradually crushed, the load decreases while the deflection continues to increase, and the curve enters the descending branch until the end of the test. The desert sand replacement ratio has a significant effect on the ultimate bearing capacity and failure deflection of DSCBs, and the specific data are shown in Table 6 and Table 7.

As *r* increases from 0% to 20%, both the ultimate load capacity and failure deflection of the specimens increase. Among them, the DSCB with *r* = 20% exhibits the highest ultimate load capacity and failure deflection under all freeze–thaw cycles, indicating that an appropriate amount of desert sand is beneficial for improving the load-bearing capacity and deformation ability of the specimens [14]. This is because an appropriate amount of desert sand can optimize the aggregate gradation, fill the voids between sand particles, improve structural compactness, and enhance the interfacial bonding between aggregates and the cement paste. When *r* increases to 40% and 60%, the performance decreases to varying degrees in most cases. This indicates that, at high replacement ratios, desert sand has a smooth surface and poor gradation, and under the same water content, the workability (slump) of the concrete mixture is lower than that of river sand concrete. This may lead to reduced compaction during casting and vibration, thereby adversely affecting the mechanical properties of concrete. In addition, the wrapping ability of the paste around aggregates is weakened, resulting in increased internal porosity and deterioration of the interfacial transition zone [26,27].

From the perspective of performance evolution under freeze–thaw conditions, the above findings have two implications. First, under non-freeze–thaw conditions, a 20% desert sand replacement ratio exhibits the best flexural performance. After freeze–thaw cycles, the advantage of the 20% replacement ratio is maintained and even enhanced (for example, after 75 cycles, the ultimate load capacity increases by 5.3% and the failure deflection increases by 37.5% compared with ordinary concrete beams), indicating that an appropriate amount of desert sand can mitigate freeze–thaw damage to a certain extent. Second, for specimens with high replacement ratios (40% and 60%), the failure deflection shows negative growth after 25 freeze–thaw cycles, indicating that they are more sensitive to freeze–thaw damage and should be used with caution in cold-region engineering. Unlike previous studies [14,26,27], which mainly focused on mechanical properties at normal temperature, this study further reveals the influence mechanism and variation law of the desert sand replacement ratio on the flexural performance of DSCBs under freeze–thaw conditions, providing a reference for the application of desert sand concrete in cold-region engineering.

#### 3.4.2. Effect of Freeze–Thaw Cycle Number

**Figure 10:**
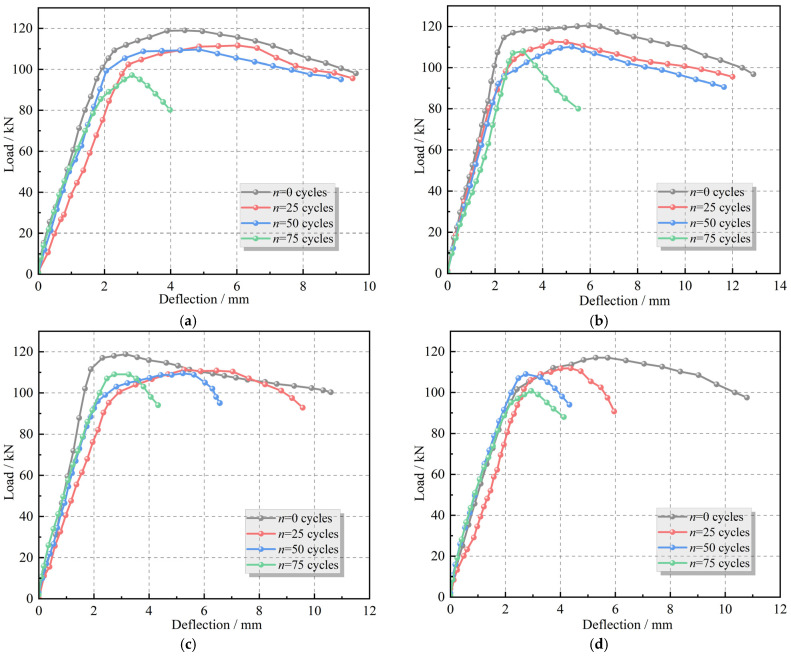
Load–deflection curves with varying freeze–thaw cycles: (**a**) *r* = 0%; (**b**) *r* = 20%; (**c**) *r* = 40%; (**d**) *r* = 60%.

As shown in Figure 10, when *r* remains constant, as *n* increases, the ultimate load and failure deflection of all specimens gradually decrease. After 75 freeze–thaw cycles, the ultimate load of ordinary concrete decreases by 17.5%, while a decrease in failure deflection from 9.6 mm to 4.0 mm is observed, representing a 58.3% drop. For DSCBs with *r* = 20%, the ultimate load decreases by 9.3%, while a decrease in failure deflection from 12.9 mm to 5.5 mm is observed, representing a 57.4% drop. For DSCBs with *r* = 40%, the ultimate load decreases by 13.2%, while a decrease in failure deflection from 10.6 mm to 4.4 mm is observed, representing a 58.5% drop. For DSCBs with *r* = 60%, the ultimate load decreases by 13.7%, while a decrease in failure deflection from 10.8 mm to 4.1 mm is observed, representing a 62.0% drop.

It can be seen that freeze–thaw cycles weaken the ultimate load and failure deflection of the specimens, whereas the DSCB with *r* = 20% exhibits the smallest reductions in ultimate load and failure deflection under the freeze–thaw environment, indicating that DSCBs with a replacement ratio of 20% has the best deformation capacity and freeze–thaw resistance. In addition, as *n* increases, the failure deflection of DSCBs gradually decreases, which is consistent with the conclusion reported in the literature [25]. The main reason is the continuous accumulation of freeze–thaw damage. At the initial stage of freeze–thaw cycling, only a small number of microcracks initiate inside the concrete, which has a limited effect on the overall structural performance. With the increase in freeze–thaw cycles, these microcracks continue to propagate and connect, resulting in significant reductions in the elastic modulus and compressive strength of the concrete, and ultimately leading to decreases in the load-carrying performance and failure deflection of the structure [28,29].

From the perspective of engineering applications, the above findings have the following two implications. First, the DSCB with *r* = 20% shows smaller reductions in ultimate load capacity (9.3%) and failure deflection (57.4%) compared with ordinary concrete beams and specimens with other replacement ratios. This indicates that an appropriate amount of desert sand can effectively mitigate the adverse effects of freeze–thaw damage on the mechanical performance of beams, which provides a reference for the design of concrete structures in cold regions. Second, even at the optimal replacement ratio, the reduction in failure deflection reaches 57.4% after 75 freeze–thaw cycles. This indicates that freeze–thaw damage weakens the deformation capacity much more significantly than the load-bearing capacity, and the deformation performance of structural members should be given special attention in frost-resistant design. The above trend is consistent with the findings of reference [25], which reported that freeze–thaw damage leads to a reduction in the deformation capacity of concrete beams. Furthermore, this study quantifies the differences in performance degradation under different desert sand replacement ratios, filling the research gap in the flexural performance of desert sand concrete beams under freeze–thaw conditions.

### 3.5. Analysis of Load–Longitudinal Reinforcement Strain Curves

#### 3.5.1. Effect of Desert Sand Replacement Ratio

**Figure 11:**
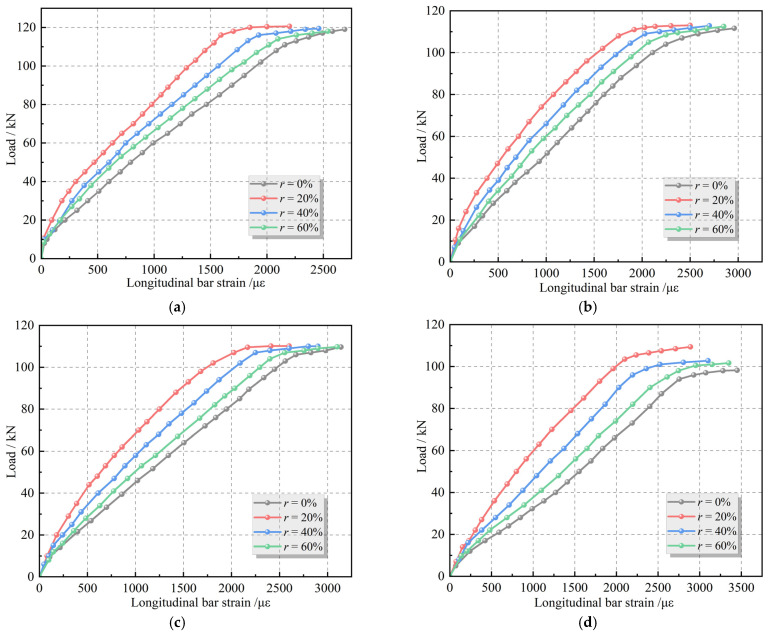
Load–longitudinal reinforcement strain curves with varying desert sand replacement ratios: (**a**) *n* = 0 cycles; (**b**) *n* = 25 cycles; (**c**) *n* = 50 cycles; (**d**) *n* = 75 cycles.

Figure 11 illustrates the variation in tensile reinforcement strain with load in DSCBs under different desert sand replacement ratios. At the initial loading stage, no cracks appear in the specimens, and the reinforcement strain increases approximately linearly with the load. After reaching the cracking load, cracks form at the bottom of the beam, and the growth rate of reinforcement strain increases significantly. As the load continues to increase until the reinforcement yields, a clear turning point appears in the load–strain curve. The slope decreases significantly and tends to flatten, with slow load growth but rapid strain increase. After reaching the ultimate load, the concrete in the compression zone is crushed, the specimen loses its load-bearing capacity, and the reinforcement strain reaches its peak. The desert sand replacement ratio has a significant effect on the longitudinal reinforcement strain of DSCB, and the detailed data are presented in Table 8.

Overall, the strain of longitudinal reinforcement in DSCBs is lower than that measured in conventional concrete specimens. Among them, DSCBs with *r* = 20% exhibit the minimum longitudinal reinforcement strain and the largest reduction under all freeze–thaw cycle conditions, indicating that an appropriate amount of desert sand is beneficial to slowing down the development of longitudinal reinforcement strain and improving the overall stiffness and cooperative mechanical behavior of the specimens. However, when the replacement ratio increases to 40% and 60%, the reduction in strain becomes less significant, indicating that the tensile coordination inside the specimens decreases at higher replacement ratios. The main reason is that an appropriate amount of desert sand can optimize the aggregate gradation of concrete, improve the compactness of the material, and effectively elevate the bond capability between reinforcing bars and concrete, allowing the reinforcement and concrete to work together more effectively and thus restraining the development of longitudinal reinforcement strain. However, when the desert sand replacement ratio is high, the increase in fine particle content leads to a higher internal porosity of concrete. Under freeze–thaw cycles, the material damage becomes more severe, and the bond performance between the reinforcement and concrete decreases, resulting in a weakened strain reduction effect or even an increase in longitudinal reinforcement strain [30,31].

From the perspective of engineering applications, the above findings have two implications. First, the DSCB with *r* = 20% maintains a stable reduction range of longitudinal reinforcement strain between 15.5% and 18.2% under different freeze–thaw cycles. This indicates that an appropriate amount of desert sand can significantly improve the stiffness reserve and coordinated load-bearing capacity of beams under freeze–thaw conditions, providing a reference for the durability design of concrete structures in cold regions. Second, for specimens with high replacement ratios (40% and 60%), the reduction in longitudinal reinforcement strain decreases to 10.1% and 2.9%, respectively, after 75 freeze–thaw cycles. The beneficial effect is almost lost, indicating that there exists an optimal threshold for desert sand replacement ratio, beyond which the freeze–thaw resistance advantage decreases sharply. Unlike previous studies [30,31], which mainly focused on the mechanical properties and bond performance of desert sand concrete at normal temperatures, this study further reveals the influence law and microscopic mechanism of desert sand replacement ratio on the longitudinal reinforcement strain of DSCBs under freeze–thaw cycles, filling the research gap in the flexural performance of desert sand concrete beams under freeze–thaw conditions.

#### 3.5.2. Impact of the Number of Freeze–Thaw Cycles

**Figure 12:**
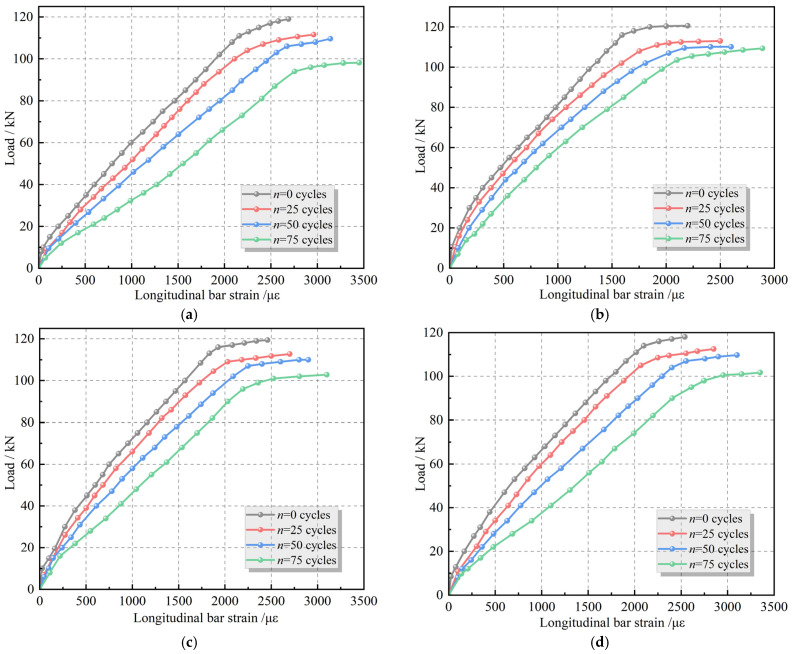
Load–longitudinal reinforcement strain curves with varying freeze–thaw cycles: (**a**) *r* = 0%; (**b**) *r* = 20%; (**c**) *r* = 40%; (**d**) *r* = 60%.

Figure 12 illustrates the variation in tensile reinforcement strain with load in DSCBs under different numbers of freeze–thaw cycles. The number of freeze–thaw cycles has a significant effect on the longitudinal reinforcement strain of DSCBs, and the detailed data are presented in Table 9.

It is apparent that, with an equal *r*, the longitudinal reinforcement strain undergoes a continuous upward trend with the increase in *n*. Specifically, after 25 freeze–thaw cycles, the increase in longitudinal reinforcement strain is relatively small, approximately 10–14%. After experiencing 50 freeze–thaw cycles, the increase rises to 16–22%. After experiencing 75 freeze–thaw cycles, the increase further rises to 26–32%. Meanwhile, the load–longitudinal reinforcement strain curves show an overall rightward shift, while the ultimate load and slope gradually decrease, the stiffness continuously degrades, and the yield point moves downward and rightward, indicating a significant reduction in yield load. There are two main reasons for this phenomenon. First, before concrete cracking, the tensile force is jointly resisted by the concrete and the reinforcement. After freeze–thaw action, the deformation resistance of concrete decreases, the cracking load becomes smaller, and the tensile force borne by the reinforcement increases, leading to earlier yielding [32]. Second, freeze–thaw cycles cause severe damage to cement-based materials and generate a large number of random microcracks inside the material, thereby accelerating the deterioration of reinforcement performance [33,34].

From the perspective of engineering applications, the above findings have two implications. First, the aggravating effect of freeze–thaw cycles on longitudinal reinforcement strain is most significant after 75 cycles (with an increase of 26–32%). This indicates that under long-term freeze–thaw action, the stiffness of beams deteriorates severely, and sufficient attention should be paid to the freeze–thaw durability of concrete structures in cold regions in design. Second, the differences in the increase in longitudinal reinforcement strain under different desert sand replacement ratios indicate that although an appropriate amount of desert sand (20%) can suppress the development of reinforcement strain to a certain extent, this beneficial effect cannot completely offset the negative impact of freeze–thaw damage. After 75 cycles, the increase in longitudinal reinforcement strain of specimens with *r* = 20% still reaches 31.4%, which is close to that of ordinary concrete beams (28.3%). Therefore, in severe freeze–thaw environments, in addition to optimizing the mix proportion, other anti-freeze measures (such as adding air-entraining agents and improving concrete impermeability) should be combined to enhance the durability of structures. Unlike previous studies [31,32,33], which mainly focused on the influence of freeze–thaw on material-level properties, this study further reveals the quantitative influence law of freeze–thaw cycle number on the longitudinal reinforcement strain of DSCB, providing experimental support for evaluating the flexural performance of desert sand concrete beams under freeze–thaw conditions.

In summary, the number of freeze–thaw cycles and the desert sand replacement ratio both have significant effects on the flexural performance of DSCBs, including failure mode, load–deflection relationship, and load–reinforcement strain relationship. The analysis in Section 3.3 shows that, even after freeze–thaw cycles, the distribution of concrete strain along the section height at the midspan of DSCBs still approximately follows a linear relationship, that is, the plane section assumption remains valid. This provides a basic premise for establishing a flexural calculation model of DSCBs under freeze–thaw conditions based on current design theory. However, the development of internal microcracks and material degradation caused by freeze–thaw cycles lead to significant deviations when directly applying the calculation formulas of ordinary concrete beams at normal temperatures. Therefore, based on the plane section assumption, this study comprehensively considers the effects of desert sand replacement ratio and freeze–thaw cycle number, and modifies the calculation formulas for cracking moment and ultimate load capacity in current codes to establish a prediction method for the flexural performance of DSCBs under freeze–thaw conditions.

## 4. Applicability Analysis of Flexural Calculation Methods for DSCBs with Freeze–Thaw Cycling

### 4.1. Analysis of Cracking Moment

As demonstrated in Section 3.3, the plane-section assumption remains valid. To establish a formula for calculating the cracking moment of DSCBs after freeze–thaw cycles, the equation provided in the Code for Design of Concrete Structures [21] is first adopted as a reference, as follows:

(1) $$M_{\mathrm{cr}}=\frac{\gamma_{m}f_{\mathrm{tk}}I_{0}}{\left(h-y_{0}\right)}$$

where *γ*_m_ is the plastic influence coefficient of the section modulus of the concrete member and is taken as 1.55; *f*_tk_ is the standard value of tensile strength, which, according to Ref. [15], is given by $f_{\mathrm{tk}}=(0.401-00.024r)f_{\mathrm{cu}}^{0.507}$ (where *r* is the desert sand replacement ratio, 0 ≤ *r* ≤ 80%); *I*_0_ is the moment of inertia of the transformed section about its centroidal axis; *y*_0_ is the height of the compression zone; and *h* is the section height.

The experimental cracking moments of DSCBs after freeze–thaw cycles $M_{\mathrm{cr}}$ and the values calculated by Equation (1) $M_{\mathrm{cr}}^{0}$ are listed in Table 10. As shown in Table 10, the experimental cracking moments of DSCBs after freeze–thaw cycles are lower than those calculated by Equation (1), that is, $M_{\mathrm{cr}}/M_{\mathrm{cr}}^{0}$ are all less than 1. The data yield a mean of 0.818, a standard deviation of 0.080, and a coefficient of variation of 0.099. This indicates that the cracking moments calculated with reference to the current code [21] are in weak accordance with the experimental findings and hence not applicable to freeze–thawed DSCBs.

To determine the effects of *r* and *n* on the cracking moment of DSCBs, the dimensionless coefficient *α*_cr_ (relative cracking moment) is introduced to eliminate the effects of section size and the intrinsic strength of concrete [34]. The expression for *α*_cr_ is:

(2) $$a_{\mathrm{cr}}=\frac{M_{\mathrm{cr}}}{bh_{0}^{2}f_{\mathrm{tk}}}$$

where *b* is the section width of the beam; *h*_0_ is the effective section depth; $M_{\mathrm{cr}}$ is the experimental cracking moment of the beam; and *f*_tk_ is the standard value of the tensile strength of concrete.

**Figure 13:**
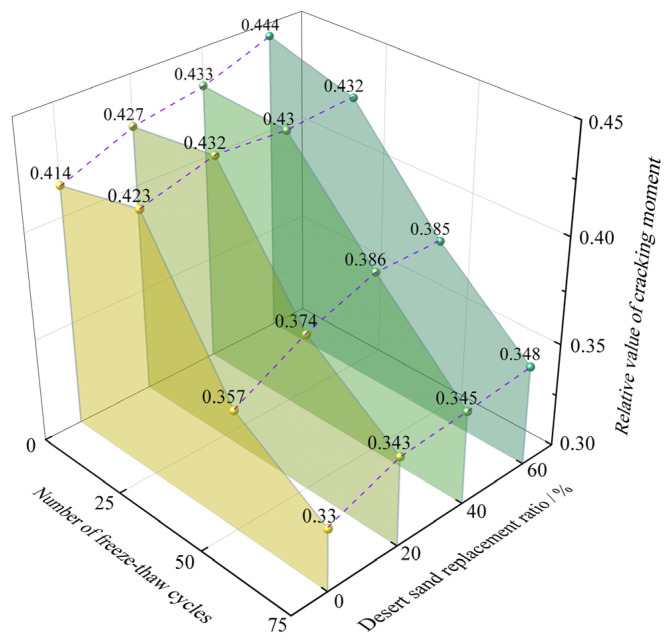
Curve of relative cracking moment variation.

Figure 13 shows the variation in the relative cracking moment of DSCBs after freeze–thaw cycles with *r* and *n.* The relative cracking moment increases with increasing *r*, but gradually decreases as *n* increases, indicating that both *r* and *n* have a certain influence on the cracking moment of DSCBs.

Based on the effects of *r* and *n* on the cracking moment of DSCBs after freeze–thaw cycles, and with reference to the calculation method in Ref. [21], a modification coefficient *k*(*r*, *n*) related to *r* and *n* is introduced to revise Equation (1). As documented in Table 10, the data yield a mean of 1.031, a standard deviation of 0.037, and a coefficient of variation of 0.036. It can be seen that the experimental cracking moments of DSCBs after freeze–thaw cycles agree well with the revised calculated values.

Regression analysis of the cracking moment values in Table 10 yields the following expression for the modification coefficient *k*(*r*, *n*) in terms of *r* and *n*, which is obtained as follows:

$$k(r,n)=0.86+0.129r-0.02n-0.042r^{2}$$

(3) $$-1.43\times 10^{-5}n^{2}+7\times 10^{-4}rn$$

In summary, the calculation formula for the cracking moment of DSCBs after freeze–thaw cycles is:

(4) $$M_{\mathrm{cr}}=k(r,n)\frac{\gamma_{m}f_{\mathrm{tk}}I_{0}}{\left(h-y_{0}\right)}$$

### 4.2. Analysis of Ultimate Bearing Capacity

The ultimate load-bearing capacity of DSCBs is determined by their failure load, and is computed in accordance with the formula provided in the Code for Design of Concrete Structures [21], as follows:

(5) $$M_{u}=a_{1}f_{c}bx(h_{0}-0.5x)$$

(6) $$a_{1}f_{c}bx=f_{y}A_{s}$$

where $a_{1}$ is the coefficient of the simplified stress block for concrete in compression and is taken as 1.0; $f_{c}$ is the compressive strength of concrete and is taken as the measured axial compressive strength of concrete; $f_{y}$ is the yield strength of reinforcement and is taken as the measured yield strength of the reinforcement; *x* is the height of the concrete compression zone; and $A_{s}$ is the effective cross-sectional area of the reinforcement.

The experimental ultimate bearing capacities of DSCBs after freeze–thaw cycles $M_{u}^{t}$ and the values calculated by Equations (5) and (6) $M_{u}^{c}$ are listed in Table 11. As observed from Table 11, the average value of $M_{u}^{t}/M_{u}^{c}$ for DSCBs after freeze–thaw cycles is 1.159, with a standard deviation of 0.042 and a coefficient of variation of 0.036, indicating that the experimental ultimate load-carrying capacities of DSCBs after freeze–thaw cycles are greater than the calculated values, with a relatively small safety margin. It can therefore be seen that the current code formula [21] for ultimate load-carrying capacity is applicable to DSCBs after freeze–thaw cycles, although its calculation accuracy still needs to be improved. To this end, a modification coefficient *η*(*r*, *n*) is introduced to revise the formula in the current code [21]. As observed from Table 11, the average value of the revised $M_{u}^{t}/M_{u}^{c1}$ is 1.040, a standard deviation of 0.033 and a coefficient of variation of 0.032. This demonstrates that the tested ultimate bearing capacities of DSCBs after freeze–thaw cycles agree well with the modified calculated values.

Regression analysis of the ultimate load-carrying capacity values in Table 11 yields the following expression for the modification coefficient *η*(*r*, *n*) in terms of *r* and *n*, which is obtained as follows:

(7) $$\eta (r,n)=1.2-9.38\times 10^{-2}r-1.87\times 10^{-3}n$$

In summary, the calculation formula for the ultimate load-carrying capacity of DSCBs after freeze–thaw cycles is:

(8) $$M_{u}=\eta (r,n)a_{1}f_{c}bx(h_{0}-0.5x)$$

## 5. Conclusions

This study investigates the flexural performance of DSCBs under freeze–thaw conditions, and the main conclusions are as follows:

(1) With the increase in *n*, the apparent damage of DSCBs gradually aggravates, mainly manifested as surface mortar spalling, fine aggregate loss, and coarse aggregate exposure. After freeze–thaw cycles, damage in stress concentration regions such as beam ends and corners becomes more significant. It is recommended to adopt additional frost-resistant structural measures for these regions in cold-region engineering.

(2) The crack development and failure modes of DSCBs are basically similar to those of ordinary concrete beams. The concrete strain at the midspan section is linearly distributed along the height, and the plane section assumption remains valid after freeze–thaw, providing a theoretical basis for the design of such members.

(3) When *r* is 20%, the DSCB exhibits the optimal comprehensive mechanical performance under all freeze–thaw cycles. It is recommended to prioritize this replacement ratio in cold-region engineering to achieve the best frost-resistant flexural performance.

(4) Freeze–thaw cycles significantly deteriorate the flexural performance of desert sand concrete beams. With increasing freeze–thaw cycles, the ultimate load capacity continuously decreases and the deformation capacity gradually weakens. Additional frost-resistant design measures are required under severe freeze–thaw conditions.

(5) By introducing correction coefficients related to the desert sand replacement ratio r and freeze–thaw cycle number n, calculation models for the cracking moment and ultimate load capacity of desert sand concrete beams under freeze–thaw conditions are established. The calculated results agree well with the experimental values, providing theoretical guidance for engineering practice.

## Figures and Tables

**Table 1:** Mix proportion of desert sand concrete.

Desert Sand Replacement Rate *r*/%	Material Consumption/(kg·m^−3^)						
	Water	Cement	Fly Ash	Superplasticizer	Coarse Aggregate	River Sand	Desert Sand
0	160	370	30	1.6	1288	552.0	0
20	160	370	30	1.6	1288	441.6	110.4
40	160	370	30	1.6	1288	331.2	220.8
60	160	370	30	1.6	1288	220.8	331.2

**Table 2:** Main physical properties of fine aggregate.

Category	Apparent Density (kg/m^3^)	Bulk Density (kg/m^3^)	Prosity (%)	Clay Content (%)	Water Ratio (%)
River sand	2038	1350	45	2.2	1.9
Desert sand	2630	1615	35	1.9	1.5

**Table 3:** Mechanical properties of concrete.

Desert Sand Replacement Rate *r*/%	Cubic Compressive Strength *f*_cu_/(N·mm^−2^)	Axial Compressive Strength *f*_c_/(N·mm^−2^)
0	44.5	21.8
20	42.7	20.9
40	41.6	20.4
60	40.3	19.8

**Table 4:** Mechanical properties of reinforcing steel.

Steel Bar Diameter *d*/mm	Yield Strength *f*_y_/MPa	Ultimate Strength *f*_u_/MPa	Elastic Modulus of Steel Bar *E*_c_/GPa
12	435	595	200
8	410	550	200
6	360	480	200

**Table 5:** Design parameters of the specimens.

Specimen	Cross-Section *b* × *h* × *l*/mm	Reinforcement Ratio *ρ*/%	Stirrups	*r*/%	*n*/Times
DSCB-1-0	100 × 150 × 800	1.51%	Φ6@50	0	0
DSCB-2-0	100 × 150 × 800	1.51%	Φ6@50	0	25
DSCB-3-0	100 × 150 × 800	1.51%	Φ6@50	0	50
DSCB-4-0	100 × 150 × 800	1.51%	Φ6@50	0	75
DSCB-1-20	100 × 150 × 800	1.51%	Φ6@50	20	0
DSCB-2-20	100 × 150 × 800	1.51%	Φ6@50	20	25
DSCB-3-20	100 × 150 × 800	1.51%	Φ6@50	20	50
DSCB-4-20	100 × 150 × 800	1.51%	Φ6@50	20	75
DSCB-1-40	100 × 150 × 800	1.51%	Φ6@50	40	0
DSCB-2-40	100 × 150 × 800	1.51%	Φ6@50	40	25
DSCB-3-40	100 × 150 × 800	1.51%	Φ6@50	40	50
DSCB-4-40	100 × 150 × 800	1.51%	Φ6@50	40	75
DSCB-1-60	100 × 150 × 800	1.51%	Φ6@50	60	0
DSCB-2-60	100 × 150 × 800	1.51%	Φ6@50	60	25
DSCB-3-60	100 × 150 × 800	1.51%	Φ6@50	60	50
DSCB-4-60	100 × 150 × 800	1.51%	Φ6@50	60	75

Note: *r* is the desert sand replacement ratio, *n* is the number of freeze–thaw cycles the same below.

**Table 6:** Variation in ultimate bearing capacity of DSCBs compared to ordinary concrete beams under different desert sand replacement ratios.

	*r*/%	20	40	60
*n*/Times				
0		+1.3%	−0.5%	−1.0%
25		+1.25%	+1.07%	+0.8%
50		+0.46%	+0.37%	+0.10%
75		+5.3%	+4.7%	+3.6%

Note: Positive values indicate an increase in DSCBs compared to ordinary concrete beams, while negative values indicate a decrease.

**Table 7:** Variation magnitude of failure deflection of DSCBs relative to ordinary concrete beams under different desert sand replacement ratios.

	*r*/%	20	40	60
*n*/Times				
0		+34.4%	+10.4%	+12.5%
25		+26.3%	+1.01%	−37.9%
50		+26.1%	−28.3%	−53.3%
75		+37.5%	−10.0%	+2.5%

Note: Positive values indicate an increase in DSCBs compared to ordinary concrete beams, while negative values indicate a decrease compared to ordinary concrete beams.

**Table 8:** Variation in longitudinal reinforcement strain of DSCBs compared to ordinary concrete beams under different desert sand replacement ratios.

	*r*/%	20	40	60
*n*/Times				
0		−18.2%	−8.6%	−5.6%
25		−15.5%	−8.8%	−3.7%
50		−17.2%	−7.6%	−1.3%
75		−16.2%	−10.1%	−2.9%

Note: The values in the table represent the decrease in longitudinal reinforcement strain of DSCBs compared to ordinary concrete beams.

**Table 9:** Variation in longitudinal reinforcement strain of DSCBs under different freeze–thaw cycles compared to DSCBs without freeze–thaw cycles.

	*r*/%	25	50	75
*n*/Times				
0		+10.0%	+16.6%	+28.3%
20		+13.7%	+18.2%	+31.4%
40		+9.8%	+17.9%	+26.0%
60		+12.2%	+22.1%	+31.9%

Note: The values in the table represent the increase in longitudinal reinforcement strain of DSCBs after freeze–thaw cycles compared to specimens without freeze–thaw cycles.

**Table 10:** Comparative analysis of calculated and experimental values of cracking moment.

Specimen	Test Value $M_{\mathrm{cr}}$/(kN·m)	Calculated Value $M_{\mathrm{cr}}^{0}$/(kN·m)	$M_{\mathrm{cr}}/M_{\mathrm{cr}}^{0}$	Calculated Value $M_{\mathrm{cr}}^{1}$/(kN·m)	$M_{\mathrm{cr}}/M_{\mathrm{cr}}^{1}$
DSCB-1-0	1.75	2.05	0.85331	1.76371	0.99223
DSCB-1-20	1.74	1.98	0.88072	1.74672	0.99615
DSCB-1-40	1.72	1.92	0.89603	1.73698	0.99022
DSCB-1-60	1.71	1.86	0.91951	1.71516	0.99699
DSCB-2-0	1.64	1.87	0.87705	1.49791	1.09486
DSCB-2-20	1.67	1.87	0.89522	1.54588	1.08029
DSCB-2-40	1.63	1.83	0.89156	1.55940	1.04527
DSCB-2-60	1.61	1.80	0.89623	1.56977	1.02562
DSCB-3-0	1.38	1.86	0.74263	1.34585	1.02538
DSCB-3-20	1.43	1.84	0.77854	1.38744	1.03067
DSCB-3-40	1.45	1.81	0.80229	1.41537	1.02447
DSCB-3-60	1.42	1.77	0.80094	1.43169	0.99183
DSCB-4-0	1.20	1.75	0.68719	1.09936	1.09154
DSCB-4-20	1.26	1.76	0.71465	1.17102	1.07599
DSCB-4-40	1.24	1.72	0.71886	1.19960	1.03367
DSCB-4-60	1.22	1.68	0.72532	1.21668	1.00273

**Table 11:** Comparative analysis of calculated and measured values of flexural capacity.

Specimen	Test Value $M_{u}^{t}$/(kN·m)	Calculated Value $M_{u}^{c}$/(kN·m)	$M_{u}^{t}/M_{u}^{c}$	Calculated Value $M_{u}^{c1}$/(kN·m)	$M_{u}^{t}/M_{u}^{c1}$
DSCB-1-0	11.90	9.98	1.19227	11.97710	0.99356
DSCB-1-20	12.06	9.89	1.21974	11.82763	1.01965
DSCB-1-40	11.84	9.83	1.20495	11.57010	1.02333
DSCB-1-60	11.78	9.75	1.20826	11.29700	1.04275
DSCB-2-0	11.16	9.57	1.16572	11.18427	0.99783
DSCB-2-20	11.30	9.63	1.17281	11.07527	1.02029
DSCB-2-40	11.28	9.61	1.17402	10.86406	1.03829
DSCB-2-60	11.25	9.59	1.17257	10.66859	1.05450
DSCB-3-0	10.96	9.55	1.14736	10.71300	1.02306
DSCB-3-20	11.01	9.57	1.15089	10.54938	1.04366
DSCB-3-40	11.00	9.56	1.15069	10.36228	1.06154
DSCB-3-60	10.97	9.54	1.15012	10.16019	1.07970
DSCB-4-0	9.82	9.22	1.06550	9.90524	0.99139
DSCB-4-20	10.94	9.35	1.16935	9.87767	1.10735
DSCB-4-40	10.28	9.31	1.10391	9.65901	1.06429
DSCB-4-60	10.17	9.25	1.09923	9.42284	1.07929

## Data Availability

The original contributions presented in this study are included in the article. Further inquiries can be directed to the corresponding authors.
